# Prevalence of *S. aureus* and/or MRSA from seafood products from Indian seafood products

**DOI:** 10.1186/s12866-022-02640-9

**Published:** 2022-10-01

**Authors:** Gopalan Krishnan Sivaraman, Sobin Sonu Gupta, S Visnuvinayagam, T Muthulakshmi, Ravikrishnan Elangovan, Vivekanandan Perumal, Ganesh Balasubramanium, Tushar Lodha, Amit Yadav

**Affiliations:** 1 Fermentation and Biotechnology Division, Indian Council of Agricultural Research- Central Institute of Fisheries Technology, Microbiology, Matsyapuri Post, Willingdon Island, Cochin, 682 029 Kerala India; 2 Department of Biochemical Engineering & Biotechnology, Indian Institute of Technology, Hauz Khas, New Delhi, 110 016 India; 3 Kusuma School of Biological Sciences, Indian Institute of Technology, Hauz Khas, New Delhi, 110 016 India; 4 Indian Council of Medical Research- National Institute of Epidemiology, Ayapakkam Chennai, 600077 India; 5 National Centre for Cell Science, NCCS Complex, Savitribai Phule University of Pune Campus, Ganeshkhind Pune, 411 007 India

**Keywords:** Antibiotic resistance, Coagulase positive staphylococci, Methicillin-resistant, Multidrug-resistant, Seafood, Whole genome sequence

## Abstract

Compared to the clinical sector, the prevalence of methicillin-resistant *Staphylococcus aureus* (MRSA) in the food sector is relatively low. However, their presence in seafood is a significant public health concern. In India, fish and fishery products are maximally manually handled compared to other food products. In this study, 498 fish samples were collected under various conditions (fresh, chilled or dressed) and representatives from their surroundings. These samples were screened for the prevalence of *Staphylococcus aureus*, determining its antimicrobial resistance, MRSA and genetic profile. It is observed that 15.0% and 3.0% of the total samples were screened positive for *S. aureus* and MRSA, respectively. The *S. aureus* strain MRSARF-10 showed higher resistance to linezolid, co-trimoxazole, cefoxitin, ofloxacin, gentamicin, rifampicin, ampicillin/sulbactam and Piperacillin–tazobactam. This MRSA, *spa* type t021 and SCC*mec* type V strain isolated from dried ribbon fish (Family *Trachipteridae*) carried virulence factors for exoenzymes such as aureolysin, serine, toxin genes and a novel MLST ST 243, as revealed from its draft-genome sequence. This highly pathogenic, multidrug-resistant and virulent *S. aureus* novel strain is circulating in the environment with chances of spreading among the seafood workers and the environment. It is further suggested that Good Hygienic Practices recommended by World Health Organization need to be followed during the different stages of seafood processing to provide pathogen-free fish and fishery products to the consumers.

**Supplementary Information:**

The online version contains supplementary material available at 10.1186/s12866-022-02640-9.

## Introduction

*Staphylococcus aureus* (*S. aureus*) is commonly found asymptomatic in the human skin and noses of 25% of healthy people and animals. Methicillin-resistant *Staphylococcus aureus* is common in a hospital environment; it has been reported in fish and fishery products [1, 2]. The staphylococcal infection rate is still higher in India because of the moderately warm and humid climate [3]. The existence of heat-stable preformed staphylococcal enterotoxins is the most common foodborne disease worldwide [4, 5]. Because of the presence of staphylococcal enterotoxins (SEs): SEA to SEE, SEG to SEI, SER to SET, *S. aureus* is regarded as one of the potential foodborne pathogens. [6, 7]. Staphylococcal enterotoxins (SEs) are associated with foodborne diseases, which cause vomiting and diarrhoea. The toxins are secreted in food by enterotoxigenic *S. aureus* strains, are heat-stable, and do not degrade even when cooked [8]. The SEs are superantigens that cause T-cell activation and proliferation; their mechanism of action is likely to entail cytokine release and cell death via apoptosis, as well as the potentially fatal toxic shock syndrome. [9, 10]. Staphylococcal infections have been generally treated with commonly used antimicrobials against Gram-positive bacteria with the choice of beta-lactam antibiotics either alone or with aminoglycosides [11]. Antibiotic use has resulted in an exponential increase in the incidence of antibiotic resistance, and multi-drug resistance strains have emerged, making eradication more challenging and threatening effective prevention and treatment. Penicillin, cephalosporins, carbapenem and other beta- lactam antibiotics may not be work effectively against MRSA treatment [12]. MRSA is one of the nosocomial pathogens causing significantly higher morbidity and mortality [13]. MRSA was only recognized as a concern in India in the 1990s after it arose in the 1980s [14]. MRSA is found in 25% of Indians in the western section of the country [15], 50.18% in Central India [16] and 70% in South India [17]. Furthermore, in Asian countries, the prevalence rate of MRSA in hospitals is 41% in India, 42% in Pakistan, 18% in the Philippines, 38% in Malaysia, 50–70% in Korea, 53–83% in Taiwan, and 70% in Hong Kong and Japan [18–20].

MRSA-contaminated food can be a severe health risk to consumers, and this resistance can be transmitted to the consumers [21, 22]. Since marine water is devoid of *S. aureus*, the presence of *S. aureus* is due to a post-harvest contamination [23]. Recently, few reports have been available regarding MRSA in fish and fishery products [24]. The screening of fish and fishery products is vital to understanding the prevalence status, MRSA profiling, spa typing and virulence gene profiling. In light of this, a study was conducted in Gujarat, India, to track the prevalence of MRSA, antibiotic resistance profiles, and virulence genes of MRSA in fish and fisheries products.

## Materials and methods

### Collection of seafood samples

A total of 498 seafood samples, including ice and water from varying sources, were collected from the fish market and processed seafood from the fish processing Industries consisting of 108 fresh (raw), 79 chilled, 64 frozen and 124 processed fish samples and 76 water and 47 ice. The study was carried out from 2012 to 2017 in the Veraval region, Gujarat state, India, to monitor the existence of MRSA in different seafood and its associated environmental samples.

### Isolation and identification of *S. aureus*

Fish samples were processed according to ISO 6888–1 and ISO 6888–2:2003 (ISO, 2003) to isolate *S. aureus* on Baird Parker Agar (Difco, USA) [25]. *S. aureus* colonies with distinct characteristics were selected for coagulase tests. The MRSA isolates were confirmed using MRSA plates from HiCrome MeReSa (HiMedia, Mumbai) and BBL CHROM agar MRSA II (Difco, USA).

### Multiplex PCR for rapid confirmation of *S. aureus* and MRSA

Multiplex PCR was used to detect *S. aureus* and MRSA [26]. Since the presence of the nuc gene (320 bp), which encodes *S. aureus* thermostable nuclease, is required for *S. aureus* confirmation. The mecA gene (278 bp), which is used to identify MRSA, is a gold standard for confirmation. For the identification of Staphylococcus genes, a 16SrRNA primer unique to Staphylococcus genes has also been included (750 bp). The GenElute Bacterial Genomic DNA Kit was used to isolate DNA (Sigma- Aldrich, Spain;). Initially, the monoplex PCR with each primer separately was carried out for initial standardization then gene amplification for DNA sequencing purposes. The reference strain’s DNA of *S. aureus* ATCC 43,300 (MRSA) and ATCC 25,923 (MSSA) were tested for positive and negative control. Multiplex PCR was carried out as per Al-Talib et al*.,* Protocol [26]. The reaction was carried out using an Agilent SureCycler 8000 (USA) with an initial denaturation at 94 °C (3 min), followed by 34 cycles of denaturation at 94 °C (30 s), annealing at 60 °C (30 s), extension at 72 °C (30 s), and a final extension at 72 °C (30 s) (5 min). The amplified PCR products were seen in a Gel Doc (BioRad, USA) under UV illumination in submerged electrophoresis with 1.5% agarose and ethidium bromide.

### Antimicrobial susceptibility testing

The Minimum Inhibitory Concentration (MIC) was performed according to CLSI recommendations [27] using BD Phoenix™ M50 Automated Microbial Identification and Antimicrobial Susceptibility Testing System using Gram Positive bacteria Combo Panel (PMIC/ID- 55. Initiall antuimicrobial sensitivity tests was carriedout on Mueller Hinton agar with Dodeca Staphylococci-1 and 2 (HiMedia, Mumbai) [11]. The inhibition zones were measured to find the sensitivity and resistance. In this investigation, the reference strains of *S. aureus* ATCC 25,923 and ATCC 43,300 were employed.

### Genome sequencing of MRSA and Genome Analysis

The bacterial genomic DNA was obtained using an isolation kit (Sigma-Aldrich, France), and the DNA quality was verified using the NanoDrop spectrophotometer and Qubit Fluorometer (Thermo, USA). The paired-end Whole Genome Sequencing (WGS) was performed in Illumina HiSeq 2500 (paired end). The number of paied- end reads was approximately 7 billion short- read sequences in pairs of ~ 300 bp, the number of bases (Mb) was 1447.5, and there was 35.11% G + C content. The FastQC v0.11.9 quality check was performed, and low-quality reads (Phred score < 20) were trimmed using cutadapt 2.8. [28, 29]. SPAdes version 3.3.15 was used to do de novo assembly of trimmed readings [30]. Further, the generated contigs were annotated using Prokka 1.14.6, and the Kyoto Encyclopaedia of Genes and Genomes database (KEGG) was utilized for the annotation and classification of metabolic pathways. and BLASTX program for comparison with the NCBI database [31, 32]. The NCBI database was used for organism annotation, gene and protein annotation, gene ontology, and pathway annotation. The complete genome sequence of the reference (CP000253.1 *Staphylococcus aureus* subsp. aureus NCTC 8325) strain was obtained from a public database (NCBI). The variants were annotated using the pipeline comprising bwa version 0.7.17-r1188 for indexing and mapping to reference genome, samtools 1.10 and bcftools 1.10.2 for variant calling [33, 34]. The spa typing of Sanger sequence [35], and virulenceFinder 2.0, abricate 1.0.1 (Github), and MLST analysis by MLST 2.19.0 and by comparing the MRSA WGS, MLST Typing (Center for Genomic Epidemiology (http://www.genomicepidemiology.org/) was performed. [36]. The strains relevant to clonal complexex 30 (CC30) were identified with the help of the PubMLST database. Using these sequences, a phylogenetic tree was generated and visualized using the interactive tree of Life (iTOL) [37]. The Beast 1.10.4 was used to construct the phylogenetic tree, and Figtree v1.4.4 (http://tree.bio.ed.ac.uk/software/figtree/) was employed to visualize the phylogenetic tree [37, 38]. CRISPR genes and Cas clusters were analyzed using default settings of the CRISPRCasFinder online server (https://crisprcas.i2bc.paris-saclay.fr/; [13] and the draft genome sequence was submitted in the NCBI GenBank with the accession number of NBZY00000000.

## Results and discussion

### Prevalence of *S. aureu*s and MRSA in seafood products

#### Multiplex PCR MRSA

Total of 498 samples collected were screened for the presence of *S. aureus* and MRSA (Table 1). Out of the 498 samples, 68 were found positive for the *S. aureus*. The prevalence of *S. aureus* in the fish and fish products samples in the Veraval region was 13.65%. Similarly, 15 samples were contaminated with MRSA from the total tested samples. Hence, the prevalence of MRSA in fish and fishery products is around 3% in the Veraval region. Based on the result, it has been observed that the fresh and processed fish had a higher level of *S. aureus* and MRSA. Water and ice samples had a less number of *S. aureus* (15.0%) and MRSA (3.0%). While comparing the worldwide incidences of MRSA in seafood products, variation in MRSA percentage was observed from very low to high. Similarly, a higher incidence of 30.0% and 22.2% MRSA were found in raw fish and prepared fishery foods in public hospitals in Salvador, Bahia, and Brazil, respectively [39]. At the same time, Sivaraman et al*.*, found a higher level of MRSA (50%) in the 173 market fish samples in Assam, India. Almost 60% of the shrimp aquaculture settings samples were positive for the MRSA in Kerala, India [40, 41]. However, Daniel Vazquez- Sanchez et al*.,* did not find any MRSA in fish and fishery products [42]. The variation in MRSA level is based on the hygienic food status. The contaminations could either form the infected person, improper hygenic practices, or poor sanitary utensils. Here, it's essential to understand that most people are asymptomatic carriers and responsible for the continuous spread of MRSA in food. There is a high possibility of MRSA transmission from fish handlers to fish and vice versa. In addition, the infected processing utensils and unhygienic environment may act as a potential source for the transmission of MRSA. Several studies on MRSA found that the hygienic conditions of food handlers are generally undesirable in most situations, such as health conditions, personal hygienic conditions, and working habits, increasing cross-contamination in processed foods [43–45]. The current findings emphasize the necessity of Good Hygiene Practices (GHP) throughout multiple processing steps, beginning with transportation and retail outlets, to limit the risk of *S. aureus* and MRSA transmission from food products to humans [46].

**Table 1:** Incidence of S*. aureus* and Methicillin-resistant *S. aureus* MRSA contamination in seafood

Fish samples	No. of Samples	*S. aureus*	MRSA by multiplex PCR *
Fresh	108	20 (18.52%)	3 (2.7)
Chilled	79	14 (17.72%)	4 (5%)
Frozen	64	6 (9.38%)	2 (3.13%)
Processed fish	124	22 (17.74%	6 (4.84%)
Water	76	4 (5.26%)	0 (0%)
Ice	47	2 (4.26%)	0 (0%)
**Total**	**498**	**68 (13.65%)**	**15 (3.01%)**

#### Antimicrobial resistance patterns of MRSA 10 strain

The antimicrobial susceptibility pattern and minimum inhibitory concentration (MIC) were carried out for these 15 number of MRSA isolates using BD Phoenix^M50^ automated bacterila identification and AST system (BD, US). The resistant pattern was varied between the type of samples collected; processed fish (6), chilled (4), fresh (3), and frozen samples (2) and there were no MRSA isolates in the water and ice samples which revealed the chance from the workers (Table 1). Thses MRSA isolates possess a higher level of resistance to erythomicin (> 4 µg/ml), gentamycin (> 8 µg/ml), norfloxacin (> 8 µg/ml), oxacillin (> 2 µg/ml), and trimethptim/ sulfamethoxazole (> 4/76 µg/ml) (Table 2). Whereas it wasis sensitive to clindamycin (< = 0.25), daptomycin (< = 0.5), levofloxacin (> 2), lincomycin (> 2), moxifloxacin (> 1), nitrofurantoin (< = 16), rifampicin (< = 0.5), teicoplanin (< = 0.5), tetracycline (< = 0.5) and vancomycin (< = 1).

**Table 2:** Antimicrobial susceptibility and Minimum Inhibitory Concentration (MIC) of MRSA isolates from the fish and fishery products

Isolate ID	MIC and susceptibility pattern															PCR *mecA* +
	**Clind**	**Dapt**	**Ery**	**Gen**	**Lev**	**Lin**	**Moxi**	**Nit**	**Nor**	**Oxa**	**Rif**	**Tei**	**Tetra**	**Trim/Sul**	**Van**	
1	< = 0.25	< = 0.5	> 4	> 8	> 2	2	> 1	< = 16	> 8	> 2	< = 0.5	< = 1	< = 0.5	> 4/76	< = 1	Yes
	S	S	R	R	S	S	S	S	R	R	S	S	S	R	S	
2	< = 0.25	< = 0.5	> 4	< = 2	> 2	< = 1	> 1	< = 16	> 8	> 2	< = 0.5	2	2	< = 1/19	< = 1	Yes
	S	S	R	S	X	S	X	S	R	R	S	S	S	S	S	
3	< = 0.25	< = 0.5	> 4	> 8	> 2	< = 1	1	< = 16	> 8	> 2	< = 0.5	2	> 8	> 4/76	< = 1	Yes
	S	S	R	R	X	S	S	S	R	R	S	S	R	R	S	
4	< = 0.25	< = 0.5	> 4	> 8	> 2	2	> 1	< = 16	> 8	> 2	< = 0.5	< = 1	< = 0.5	> 4/76	< = 1	Yes
	S	S	R	R	X	S	X	S	R	R	S	S	S	R	S	
5	< = 0.25	< = 0.5	> 4	> 8	> 2	2	> 1	< = 16	> 8	> 2	< = 0.5	< = 1	< = 0.5	> 4/76	< = 1	Yes
	S	S	R	R	X	S	X	S	R	R	S	S	S	R	S	
6	< = 0.25	< = 0.5	> 4	> 8	> 2	2	> 1	< = 16	> 8	> 2	< = 0.5	< = 1	< = 0.5	> 4/76	< = 1	Yes
	S	S	R	R	X	S	X	S	R	R	S	S	S	R	S	
7	< = 0.25	< = 0.5	> 4	> 8	> 2	2	> 1	< = 16	> 8	> 2	< = 0.5	< = 1	< = 0.5	> 4/76	< = 1	Yes
	S	S	R	R	X	S	X	S	R	R	S	S	S	R	S	
8	< = 0.25	< = 0.5	> 4	> 8	> 2	2	> 1	< = 16	> 8	> 2	< = 0.5	< = 1	< = 0.5	> 4/76	< = 1	Yes
	S	S	R	R	X	S	X	S	R	R	S	S	S	R	S	
9	< = 0.25	< = 0.5	> 4	> 8	> 2	2	> 1	< = 16	> 8	> 2	< = 0.5	< = 1	< = 0.5	> 4/76	< = 1	Yes
	S	S	R	R	X	S	X	S	R	R	S	S	S	R	S	
10	< = 0.25	< = 0.5	> 4	> 8	> 2	2	> 1	< = 16	> 8	> 2	< = 0.5	< = 1	< = 0.5	> 4/76	< = 1	Yes
	S	S	R	R	X	S	X	S	R	R	S	S	S	R	S	
11	< = 0.25	< = 0.5	> 4	> 8	> 2	2	> 1	< = 16	> 8	> 2	< = 0.5	< = 1	< = 0.5	> 4/76	< = 1	Yes
	S	S	S	R	X	S	X	S	R	R	S	S	S	R	S	
12	< = 0.25	< = 0.5	> 4	> 8	> 2	2	> 1	< = 16	> 8	> 2	< = 0.5	< = 1	< = 0.5	> 4/76	< = 1	Yes
	S	S	R	R	X	S	X	S	R	R	S	S	S	R	S	
13	< = 0.25	< = 0.5	> 4	> 8	> 2	2	> 1	< = 16	> 8	> 2	< = 0.5	< = 1	< = 0.5	> 4/76	< = 1	Yes
	S	S	R	R	X	S	X	S	R	R	S	S	S	R	S	
14	< = 0.25	< = 0.5	> 4	> 8	> 2	2	> 1	< = 16	> 8	> 2	< = 0.5	< = 1	< = 0.5	> 4/76	< = 1	Yes
	S	S	R	R	X	S	X	S	R	R	S	S	S	R	S	
15	> 2	< = 0.5	> 4	> 8	> 2	> 4	> 1	> 64	> 8	> 2	1	> 16	< = 0.5	< = 1/19	> 16	*Yes*
	R	S	R	R	X	R	X	R	R	R	S	R	S	S	R	

*Clind* Clindamycin, *Dapt* Daptomycin, *Ery* Erythromycin, *Gen* Gentamicin, *Lev* Levomycin, *Lin* Lincomycin, *Mox* Moxifloxacin, *Nit* Nitrofurantoin, *Nor* Norfloxacin, *Oxa* Oxacillin, *Rif* Rifampicin, *Tei* Teicoplanin, *Tet* Tetracycline, *Trim/Sul* Trimethoprim/ sulphamethoxazole, *Van* Vacomycin

This might be due to the frequent manual handling of the fish.

Manal et al. recommended Cefoxitin as a marker for detecting methicillin resistance and they found 16- 60% of isolates were showed varied level of resistance to cefoxitin from the Riyadh hospital armed forces hospital clinical samples.Further they suggested that Cefoxitin could be considered a surrogate marker for the detection of MRSA [47].

#### Whole-genome sequence analysis of Novel MRSA ST243 strain by annotation, gene ontology and pathway analysis, rRNA genes

**Fig. 1:**
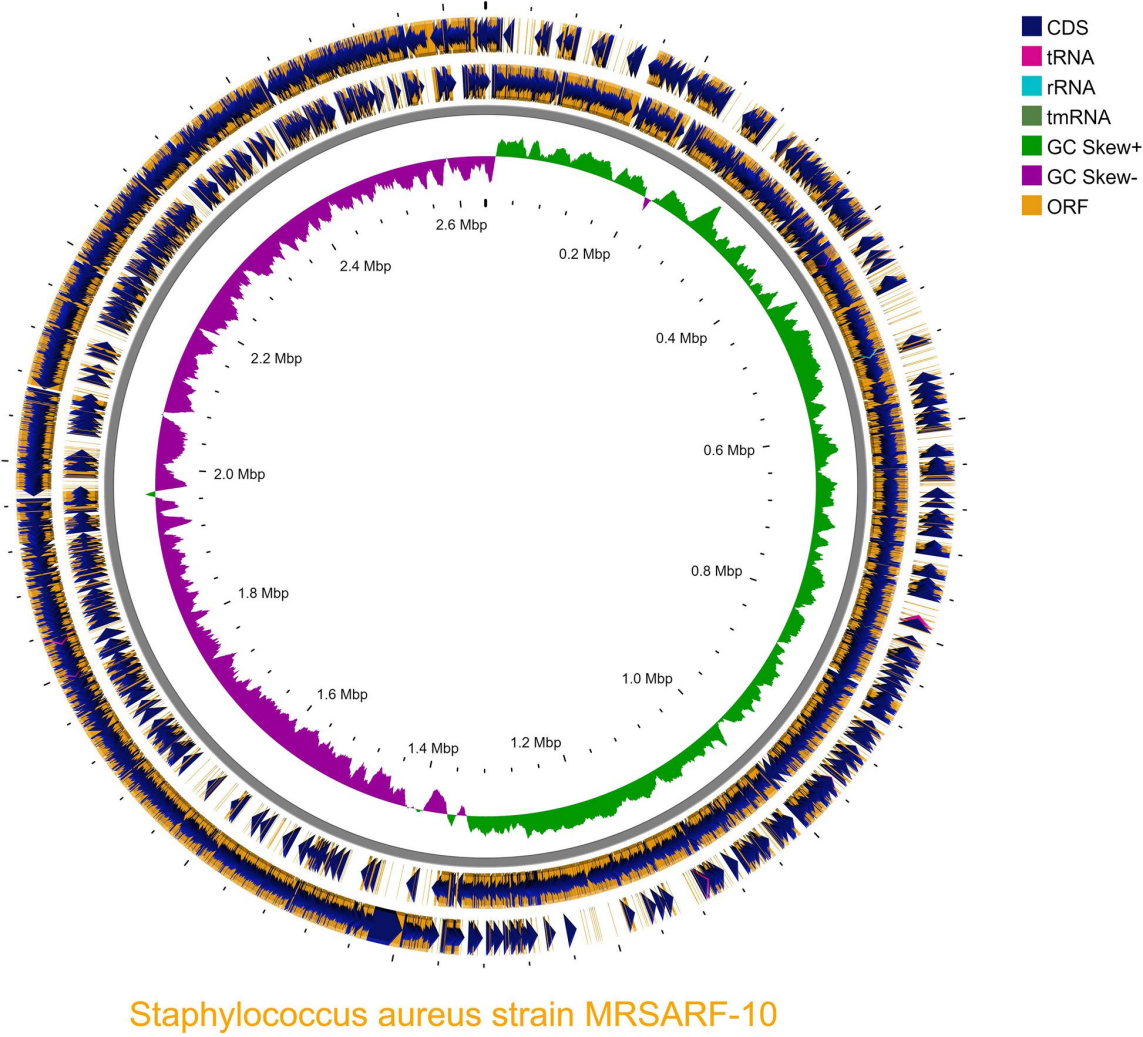
Graphical circular genome map of Methicillin-Resistant *Staphylococcus aureus* 10. From outside to the Centre: Genes on the forward strand, genes on the reverse strand, GC skew [18]

**Fig. 2:**
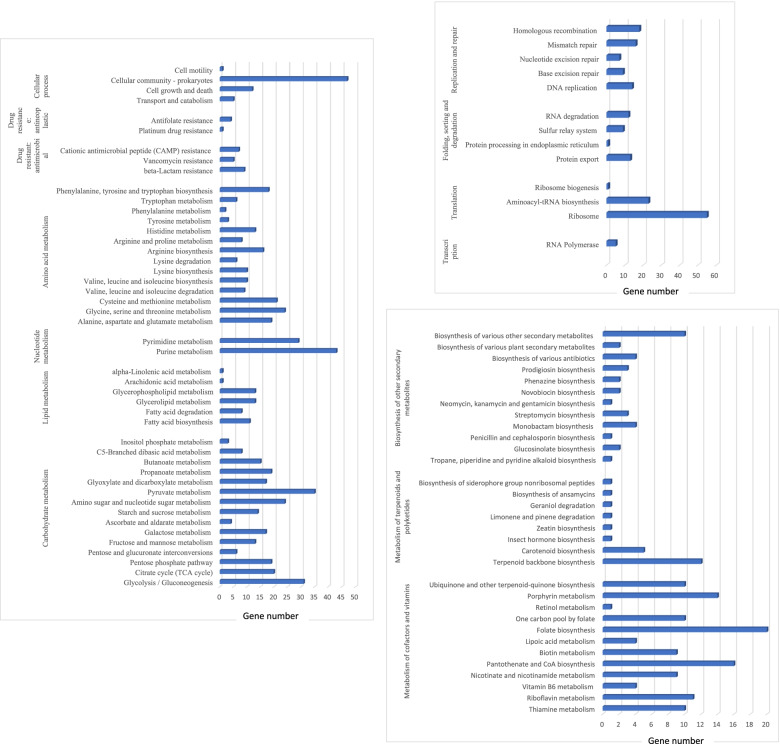
Kyoto Encyclopedia of Genes and Genome ortholog (KEGG-ortholog) Functional annotation of MRSA ST243 strain. Detailed representation of functional classes belonging to Cellular process, Antibiotic resistance, Amino acid metabolism, Nucleotide metabolism, Lipid metabolism, carbohydrate metabolism, genetic information processing, Metabolism of cofactors, metabolism of terpenoids and polyketides, Biosynthesis of other secondary metabolites

**Fig. 3:**
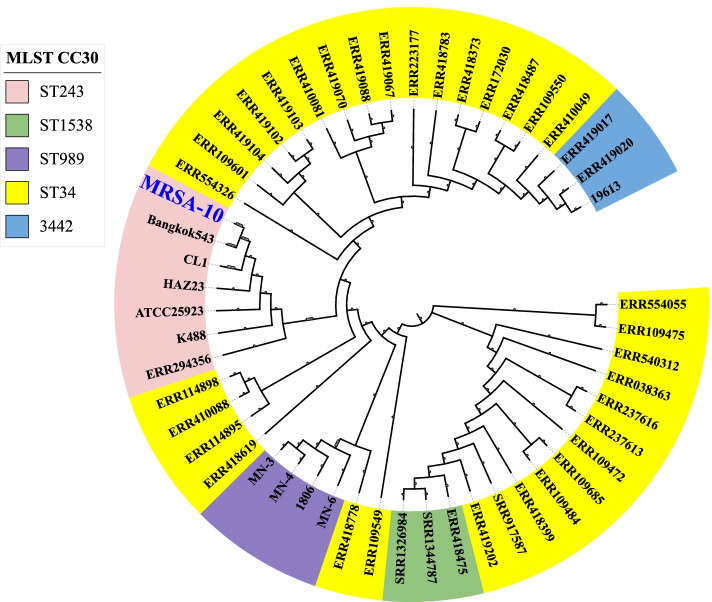
Phylogenetic analysis of the MRSA-10 strain with the relevant CC30 sequence types

The Paired-end sequencing of the MRSA isolate performed was later subjected to a quality check by Fastqc and trimmed to generate high-quality raw reads with phread score ≥ 30 excluding adaptor sequences (WGS information in supplementary table 1a). Appx. 6,157,589 raw reads with 92.87 average long reads with 164.27 × of average coverage and 3,079,132 total nucleotides with 34% GC content. De novo contigs and scaffolds were generated using Spades, wherein 158 contigs and 136 scaffolds were identified, and a quality check was performed using the Quast against the reference sequence (CP000253.1) confirming the number of contigs to be 158 with GC% of 32.79%, 2,397,067 total aligned length and 64,451 bp N50 (the details furnished in the supplementary table 1b). Furthermore, annotation with prokka revealed that the MRSA-10 contains a 2,637,041 bases long genome, 2411 CDS, 2424 genes, 2424mRNA, 1 rRNA, 11 tRNA and 1 tmRNA (Fig. 1). On further analysis with the BLASTX, the first 15 organisms hit were in correspondence with Staphylococcus species, and appx. 62.8% of genes i.e. 1537 out of 2449 genes were annotated, providing information related to various function categories in the KEGG pathway (Fig. 2). The total number of Gene Ontology annotations identified for molecular functions was 870, with 586 annotations having to do with a biological process and 236 annotations having to do with cellular components. The phylogenetic relatedness of MRSA isolate (NBZY00000000.1) was seen with the ST243 clusted of CC30 shown in phylogenetic tree constructed using iTOL (Fig. 3).. The variants were identified and filtered for Single Nucleotide Polymorphism (SNP) with the variant calling pipeline. In short, the variations identified were summarised in the form of two types of substitution mutation, i.e., 31,677 nt undergoing Transition mutation in which A to G and C to T transition was 16,110 and 15,567 respectively whereas, 18,869 nt undergoing transversions out of which A to C: 3821, A to T: 9778, C to G: 1404, and G to T: 3866 variations was noticed. The sorting of the CRISPR sequence by the CRISPRcasFinder web server resulted in Nine CRISPR regions (Table no. 3). Using The WGS was submitted at NCBI GenBank with the accession number of NBZY00000000 [48]. Overall, 98.57% (2,424 CDs) were predicted with at least one hit in the NCBI database, and 100% of the predicted CDSs have a similarity of more than 60% at the protein level in the NCBI database.

**Table 3:** De novo CRISPR/Cas prediction with the help of CRISPRCasFinder

CRISPR_Id	CRISPR_Start	CRISPR_End	CRISPR_Length	Potential_Orientation (AT%)	Consensus_Repeat	Repeat_ID (CRISPRdb)	Conservation_Repeats (% identity)	Repeat_Length	Evidence_Level
SRR538_1	682,402	682,501	99	Unknown	AAGAGCCCCTAATTAATAAATTAAAAGGGG	R271	100	30	1
SRR538_2	768,363	768,447	84	Forward	CACCCCAACTTGCATTGTCTGTAGAA	R1692	96.1	26	1
SRR538_3	826,136	826,215	79	Reverse	CCGTCAGCTTCTGTGTTGGGGCCC	R2322	95.8	24	1
SRR538_4	875,258	875,343	85	Unknown	AAAGTCAGCTTACAATAATGTGCAAGTTGG	Unknown	96.6	30	1
SRR538_5	1,169,979	1,170,071	92	Reverse	TAAGAAACAGTAATCAATAAATTGATAACT	R7515	100	30	1
SRR538_6	1,832,483	1,832,610	127	Unknown	AATTATGGAGCGGAAGATAGGATTTACACCTATACCTC	R441	97.3	38	1
SRR538_7	1,859,907	1,859,989	82	Forward	TCTGTGTTGGGGCCCCGCCAACCTGCA	Unknown	96.2	27	1
SRR538_8	2,058,028	2,058,117	89	Reverse	CAACTTTAGTTGTTAGGGGCTCTT	R1624	91.6	24	1
SRR538_9	2,130,159	2,130,284	125	Reverse	CCTCTTTACTCGAAAGCTCACAAAACTCTTGATATCA	Unknown	97.2	37	1

#### Virulence genes analysis of Novel MRSA ST243 strain

The MRSA virulence gene profiles were analyzed by VirulenceFinder 2.0 from a whole-genome sequence to assess the extent of its pathogenicity/ toxins production nature. The ability of staphylococci to produce cytotoxins (hemolysins, leukotoxins, and leukocidins) and superantigenic toxins are linked to their virulence (enterotoxins, exfoliative toxins, and toxic shock syndrome toxin) (Table 4). This MRSA strain contains identical virulence factors for exoenzyme genes such as aureolysin (aur) and serine protease (spIE) with sizes of 1530 and 717 bp, respectively accession numbers CP009554.1 and BX571856.1. MRSA virulence genes include gamma-hemolysin chain II precursor (hlgA), gamma-hemolysin component B precursor (hlgB), gamma-hemolysin component C (hlgC), Panton-Valentine leukocidin F component (lukF-PV), and Panton-Valentine leukocidin S component (lukF-PV), enterotoxin G (seg), enterotoxin I (sei), enterotoxin M (sem), enterotoxin N (sen), enterotoxin O (seo) and enterotoxin U (seu) with 100% identity. This MRSA strain has a diverse set of virulence factors/ toxin genes that have been identified as potential pathogen. These genes are predominantly expressed on mobile genetic elements and can be passed between strains via horizontal gene transfer. The presences of PVL (LukS and LukF proteins) are cytotoxic to different leukocytes and macrophages and other cells. PVL has been linked to community-acquired (CA)-MRSA infections and Sivaraman et al*.* found an increase in CA-MRSA infections in seafood [46]. Hemolysins, including hlgA, hlgB, and hlgC, are well-known toxins that cause cell lysis and death in blood cells whereas, Alpha hemolysin is the most potent membrane damaging toxin to blood cells, and is sensitive to platelets and monocytes lead to cell lysis and release of cytokines. Trigger the production of inflammatory mediators leads to septic shock symptoms during severe infections [48, 49]. Beta hemolysin/ b-toxin is sphingomyelinase that damages membrane rich in lipid.

**Table 4:** Detection of toxin genes in MRSA using VirulenceFinder 2.0

Virulence factor	Identity	Query/ Template length	Contig	Position in contig	Protein function	Accession number
hlgA	100	930/930	NBZY0100001.1 *Staphylococcus aureus* strain MRSA-10 MRSA10_Scaffold_1.whole genome shotgun sequence	99,768.100697	gamma-hemolysin chain II precursor	CP009554.1
hlgA	100	930/930	NBZY0100001.1 *Staphylococcus aureus* strain MRSA-10 MRSA10_Scaffold_1.whole genome shotgun sequence	99,768.100697	gamma-hemolysin chain II precursor	LN626917.1
hlgB	100	978/978	NBZY0100001.1 *Staphylococcus aureus* strain MRSA-10 MRSA10_Scaffold_1.whole genome shotgun sequence	97,292.98269	gamma-hemolysin component B precursor	BX571856.1
hlgC	100	948/948	NBZY0100001.1 *Staphylococcus aureus* strain MRSA-10 MRSA10_Scaffold_1.whole genome shotgun sequence	98,271.99218	gamma-hemolysin component C	CP009554.1
lukF-PV	100	978/978	NBZY01000018.1 *Staphylococcus aureus* strain MRSA-10 MRSA10_Scaffold_18.whole genome shotgun sequence	52,183.53160	Panton Valentine leukocidin F component	AB678716.1
lukF-PV	100	978/978	NBZY01000018.1 *Staphylococcus aureus* strain MRSA-10 MRSA10_Scaffold_18.whole genome shotgun sequence	52,183.53160	Panton Valentine leukocidin F component	HM584704.1
lukS-PV	100	939/939	NBZY01000018.1 *Staphylococcus aureus* strain MRSA-10 MRSA10_Scaffold_18.whole genome shotgun sequence	53,162.54100	Panton Valentine leukocidin S component	AB045978.2
lukS-PV	100	939/939	NBZY01000018.1 *Staphylococcus aureus* strain MRSA-10 MRSA10_Scaffold_18.whole genome shotgun sequence	53,162.54100	Panton Valentine leukocidin S component	AB256039.1
Seg	99.87	778/778	NBZY01000033.1 *Staphylococcus aureus* strain MRSA-10 MRSA10_Scaffold_33.whole genome shotgun sequence	4972.5748	enterotoxin G	CP002388.1
Sei	100	729/729	NBZY01000033.1 *Staphylococcus aureus* strain MRSA-10 MRSA10_Scaffold_33.whole genome shotgun sequence	2227.2599	enterotoxin I	CP002388.1
Sem	99.86	720/720	NBZY01000033.1 *Staphylococcus aureus* strain MRSA-10 MRSA10_Scaffold_33.whole genome shotgun sequence	1473.2192	enterotoxin M	CP002388.1
Sen	97.43	777/777	NBZY01000033.1 *Staphylococcus aureus* strain MRSA-10 MRSA10_Scaffold_33.whole genome shotgun sequence	3912.4688	enterotoxin N	AP014653.1
Seo	100	765/765	NBZY01000033.1 *Staphylococcus aureus* strain MRSA-10 MRSA10_Scaffold_33.whole genome shotgun sequence	427.1191	enterotoxin O	CP002388.1
Seu	100	786/786	NBZY01000033.1 *Staphylococcus aureus* strain MRSA-10 MRSA10_Scaffold_33.whole genome shotgun sequence	3109.3894	enterotoxin U	CP002388.1
Aur	100	1530/1530	NBZY01000017.1 *Staphylococcus aureus* strain MRSA-10 MRSA10_Scaffold_17.whole genome shotgun sequence	44,999..46528	aureolysin	CP009554.1
Sp1E	100	717/717	NBZY01000033.1 *Staphylococcus aureus* strain MRSA-10 MRSA10_Scaffold_33.whole genome shotgun sequence	13,636..14352	serine protease splE	BX571856.1

Virulence factors for secreted exoenzyme genes, including aureolysin (aur) and serine protease (spIE) destroy host compounds or disrupt host metabolic and signalling pathways. The protease aureolysin (neutral proteinase of *S. aureus*) degrades numerous proteins, including insulin B, and inactivates PSMs, resulting in osteomyelitis [50]. It also causes the maturation of glutamyl endopeptidase SspA by cleaving glutamate residues. As a result, the aureolysin, glutamyl endopeptidase, and cysteine proteases staphopain A and B interfere with complement factors, causing bacterial death to be evaded [51]. Exfoliative toxins cleave desmosomal cadherins in the superficial skin layers, causing staphylococcal scalded skin syndrome (SSSS), a severe skin illness characterized by a rash, blisters, and severe lesional damage to the skin [52].

#### Spa typing of MRSA isolates by on Sanger sequencing

The standardized nomenclature and availability of spa types on the central spa server (http://spaserver.ridom.de) allow researchers to study clonal diversity and MRSA transmission in hospitals and community settings. T021 and its repetitions were the most prevalent spa Type in the strain 15–12-16–02-16–02-25–17-24, contig position (NBZY0100001601, 29,990–30,243) and plus orientation. Sivaraman et al*.,* reported on the prevalence of CA- MRSA in seafood [1]. However, an MLST study of the complete genome sequenced (NBZY00000000.1) found that MRSA isolates in fish and fisheries products in India were typed to a new ST 243 (Table 5) with arcC, aroE, glpF, gmk, pta, tpi, ygiL genes with 100% identity and coverage with the alleles of 2, 2, 5, 2,6, 3, and 2, respectively. Type t021 represents the so-called new ST 243 MRSA, often detected in Gujarat State, India, in fish and fisheries items.

**Table 5:** Multi locus sequence typing (MLST) analysis of the whole genome sequence (NBZY00000000.1) in MRSA novel sequence type 243

Locus	Identity	Coverage	Alignment Length	Allele Length	Gaps	Allele
arcC	100	100	456	456	0	arcC_2
aroE	100	100	456	456	0	aroE_2
glpF	100	100	465	465	0	glpF_5
gmk	100	100	417	417	0	gmk_2
pta	100	100	474	474	0	pta_6
tpi	100	100	402	402	0	tpi_3
yqiL	100	100	516	516	0	yqiL_2

The present study shows that MRSA contamination occurred in the retail fish market and fish processing industrial samples; these isolates were resistant to rifampicin, cefoxitin Cefoxitin, and co-trimoxazole gentamicin, linezolid, penicillin, ofloxacin, piperacillin-tazobactam and ampicillin-sulbactam, i.e. multidrug-resistant. To ensure the delivery of safe, wholesome seafood delivery, all fish handlers should be made aware of the need for personal hygiene and sanitary handling techniques at all stages of processing, preserving the cold chain, adequate cleaning and disinfection of equipment, and preventing cross-contamination. This study emphasizes the importance of ongoing antibiotic susceptibility testing for *S. aureus* and MRSA in seafood to identify the source of contamination.

The present study revealed that 13.65% of samples were contaminated with *S. aureus,* and 3% were with MRSA. The MRSA strain has spa type t021 with a novel MLST ST 243 identified in fish and fishery products. This MRSA carries virulence factors such as aureolysin (aur) & serine and toxin genes such as hlgA, hlgB, hlgC, lukF-PV, lukS-PV, seg, sei, sem, sen, seo and seu.. So, the presence of highly pathogenic, MDR and virulent MRSA strains in the fish and fishery products could pose a severe threat to the consumers, which will guide us to design a better surveillance protocol and control measures. It further suggested that Good Hygienic Practices, as recommended by WHO, need to be followed strictly during various stages of handling and processing of fish and fishery products to provide wholesome fish to the consumers [53, 54].

## Supplementary Information

**Additional file 1:** 

## Data Availability

All of the relevant data such as raw data, samples, records and sequencing information’s (NGS) are available with the Corresponding author and will be shared on request. Please address all correspondence concerning this manuscript to me at gkshivraman@gmail.com.
