# Supplementary material for: Prevalence of S. aureus and/or MRSA from seafood products from Indian seafood products
Source: BMC Microbiol. 2022 Oct 1;22:233. doi: 10.1186/s12866-022-02640-9 (PMC9526301; doi:10.1186/s12866-022-02640-9)
Supplement: Supplementary file 1 — Additional file 1: [file 12866_2022_2640_MOESM1_ESM.docx]

**Supplementary Table 1a Whole-genome sequence project information**

| **Description** | **Value** |
| --- | --- |
| Finishing quality | Draft genome |
| Library used |  |
| Sequencing platform | Illumina HiSeq 2500 |
| Library type | Paired End |
| Project type | De novo genome assembly |
| Assembly method | Spades |
| Total sequence | 3079132 |
| Total length | 2692097 |
| N50 | 98644 |
| NG50 | 98644 |
| Genome coverage | 164.27x |
| GC content | 34 |
| MaxRead Length | 100 |
| Min Read length | 35 |
| GeneBank ID | NBZYOOOOOO |
| Bioproject ID | PRJNA352109 |
| BiSample | SAMN05969311 |
| Source | Dried Ribbonfish |

**Supplementary Table 1b**

| **Assembly** | **contigs** |
| --- | --- |
| Number of contigs (>= 0 bp) | 158 |
| Number of contigs (>= 1000 bp) | 71 |
| Number of contigs (>= 5000 bp) | 56 |
| Number of contigs (>= 10000 bp) | 51 |
| Number of contigs (>= 25000 bp) | 36 |
| Number of contigs (>= 50000 bp) | 19 |
| Total length (>= 0 bp) | 2705616 |
| Total length (>= 1000 bp) | 2681801 |
| Total length (>= 5000 bp) | 2651733 |
| Total length (>= 10000 bp) | 2614336 |
| Total length (>= 25000 bp) | 2340325 |
| Total length (>= 50000 bp) | 1745070 |
| # contigs | 84 |
| Largest contig | 189133 |
| Total length | 2690347 |
| Reference length | 2821361 |
| GC (%) | 32.79 |
| Reference GC (%) | 32.87 |
| N50 | 64451 |
| NG50 | 59376 |
| N75 | 36250 |
| NG75 | 32612 |
| L50 | 12 |
| LG50 | 14 |
| L75 | 26 |
| LG75 | 29 |
| # misassemblies | 36 |
| # misassembled contigs | 21 |
| Misassembled contigs length | 1603285 |
| # local misassemblies | 111 |
| # scaffold gap ext. mis. | 0 |
| # scaffold gap loc. mis. | 0 |
| # unaligned mis. contigs | 4 |
| # unaligned contigs | 22 + 38 part |
| Unaligned length | 288078 |
| Genome fraction (%) | 84.904 |
| Duplication ratio | 1.003 |
| # N's per 100 kbp | 0 |
| # mismatches per 100 kbp | 1880.74 |
| # indels per 100 kbp | 65.71 |
| Largest alignment | 103233 |
| Total aligned length | 2397067 |
| NA50 | 39228 |
| NGA50 | 37515 |
| NA75 | 19321 |
| NGA75 | 16493 |
| LA50 | 21 |
| LGA50 | 22 |
| LA75 | 45 |
| LGA75 | 51 |
